# 1,1′,5,5′-Tetra­methyl-2,2′-diphenyl-4,4′-[*p*-phenyl­enebis(methyl­idynenitrilo)]di-1*H*-pyrazol-3(2*H*)-one

**DOI:** 10.1107/S1600536808021028

**Published:** 2008-07-12

**Authors:** Yan Xiao, Cai Feng Bi, Ai Dong Wang, Yu Hua Fan, Xia Zhang, Jia Kun Xu, Si Tang Xie

**Affiliations:** a Key Laboratory of Marine Chemistry, Theory and Technology, Ministry of Education, College of Chemistry, Ocean University of China, Qingdao Shandong 266100, People’s Republic of China

## Abstract

In the centrosymmetric title compound, C_30_H_28_N_6_O_2_, the dihedral angles between the anti­pyrine ring and the terminal phenyl and central benzene rings are 50.55 (10) and 14.62 (9)°, respectively. Some short inter­molecular C—H⋯O inter­actions may help to establish the packing. An intramolecular C—H⋯O hydrogen bond is also present.

## Related literature

For related structures, see: Guo *et al.* (2007 ▶); Selvakumar *et al.* (2007 ▶). For bond-length data, see: Allen *et al.* (1987 ▶).
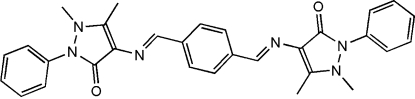


## Experimental

### 

#### Crystal data

C_30_H_28_N_6_O_2_
*M* *_r_* = 504.58
Monoclinic, 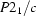

*a* = 6.0710 (2) Å
*b* = 22.2948 (7) Å
*c* = 9.8712 (3) Å
β = 95.147 (2)°
*V* = 1330.70 (7) Å^3^
*Z* = 2
Mo *K*α radiation
μ = 0.08 mm^−1^
*T* = 292 (2) K
0.18 × 0.10 × 0.09 mm

#### Data collection

Bruker APEX2 CCD diffractometer
Absorption correction: none
9162 measured reflections
3034 independent reflections
1545 reflections with *I* > 2σ(*I*)
*R*_int_ = 0.027

#### Refinement

*R*[*F* ^2^ > 2σ(*F* ^2^)] = 0.049
*wR*(*F* ^2^) = 0.141
*S* = 1.03
3034 reflections
174 parameters
H-atom parameters constrained
Δρ_max_ = 0.12 e Å^−3^
Δρ_min_ = −0.18 e Å^−3^

### 

Data collection: *SMART* (Bruker, 2001 ▶); cell refinement: *SAINT* (Bruker, 2001 ▶); data reduction: *SAINT*; program(s) used to solve structure: *SHELXS97* (Sheldrick, 2008 ▶); program(s) used to refine structure: *SHELXL97* (Sheldrick, 2008 ▶); molecular graphics: *PLATON* (Spek, 2003 ▶); software used to prepare material for publication: *SHELXTL* (Sheldrick, 2008 ▶).

## Supplementary Material

Crystal structure: contains datablocks global, I. DOI: 10.1107/S1600536808021028/hb2742sup1.cif

Structure factors: contains datablocks I. DOI: 10.1107/S1600536808021028/hb2742Isup2.hkl

Additional supplementary materials:  crystallographic information; 3D view; checkCIF report

## Figures and Tables

**Table 1:** Hydrogen-bond geometry (Å, °)

*D*—H⋯*A*	*D*—H	H⋯*A*	*D*⋯*A*	*D*—H⋯*A*
C11—H11*C*⋯O1^i^	0.96	2.36	3.321 (2)	179
C11—H11*A*⋯O1^ii^	0.96	2.47	3.375 (3)	157
C12—H12⋯O1	0.93	2.30	3.002 (2)	132

Symmetry codes: (i) 
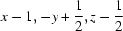
; (ii) 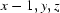.

**Fig. 1.**
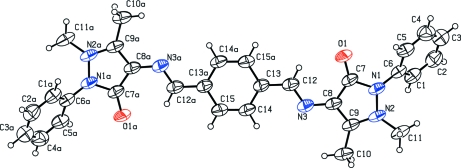
The molecular structure of (I) with displacement ellipsoids drawn at the 50% probability level for the non-hydrogen atoms. Atoms with the suffix a are generated by the symmetry operation (2-x, 1-y, 1-z).

**Fig. 2.**
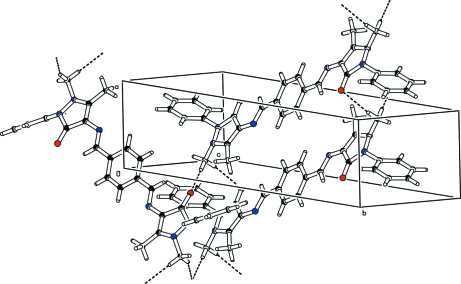
A packing diagram for (I). Hydrogen bonds are shown as dashed lines.

## supplementary crystallographic information

### Comment

Recently, some new Schiff bases of 4-aminoantipyrine have been reported (Guo *et al.*, 2007; Selvakumar *et al.*, 2007). We herein report the crystal structure of the related title compound, (I).

The complete molecule of (I) is generated by inversion and its bond lengths and angles are within normal ranges (Allen *et al.*, 1987). The maximum deviation from the mean plane for the antipyrine ring (N1/N2/C7—C9) is 0.039 (2)Å for N2. The dihedral angles between the mean planes of the antipyrine ring and the terminal and central benzene rings are 50.55 (10)° and 14.62 (9)°, respectively.

In the crystal, weak intermolecular C—H···O hydrogen bonds (Table 1) lead to chains of molecules (Fig. 2).

### Experimental

The title compound was synthesized according to the literature method (Selvakumar *et al.*, 2007). Orange plates of (I) were obtained by slow evaporation of a dichloromethane solution at 292 K.

### Refinement

All H atoms were positioned geometrically, with C—H = 0.93 and 0.96 Å for aromatic and methyl H, respectively, and constrained to ride on their parent atoms, with *U*_iso_(H) = 1.2*U*_eq_(C) or 1.5*U*_eq_(methyl C).

### Crystal data

**Table:**

C_30_H_28_N_6_O_2_	*F*_000_ = 532
*M**_r_* = 504.58	*D*_x_ = 1.259 Mg m^−^^3^
Monoclinic, *P*2_1_/*c*	Mo *K*α radiation λ = 0.71073 Å
Hall symbol: -P 2ybc	Cell parameters from 1828 reflections
*a* = 6.0710 (2) Å	θ = 2.3–22.5º
*b* = 22.2948 (7) Å	µ = 0.08 mm^−^^1^
*c* = 9.8712 (3) Å	*T* = 292 (2) K
β = 95.147 (2)º	Plate, orange
*V* = 1330.70 (7) Å^3^	0.18 × 0.10 × 0.09 mm
*Z* = 2	

### Data collection

**Table:**

Bruker APEX2 CCD diffractometer	1545 reflections with *I* > 2σ(*I*)
Radiation source: fine-focus sealed tube	*R*_int_ = 0.027
Monochromator: graphite	θ_max_ = 27.5º
*T* = 292(2) K	θ_min_ = 1.8º
ω scans	*h* = −7→7
Absorption correction: none	*k* = −28→17
9162 measured reflections	*l* = −12→9
3034 independent reflections	

### Refinement

**Table:**

Refinement on *F*^2^	Secondary atom site location: difference Fourier map
Least-squares matrix: full	Hydrogen site location: inferred from neighbouring sites
*R*[*F*^2^ > 2σ(*F*^2^)] = 0.049	H-atom parameters constrained
*wR*(*F*^2^) = 0.141	*w* = 1/[σ^2^(*F*_o_^2^) + (0.0656*P*)^2^ + 0.0153*P*] where *P* = (*F*_o_^2^ + 2*F*_c_^2^)/3
*S* = 1.03	(Δ/σ)_max_ = 0.001
3034 reflections	Δρ_max_ = 0.12 e Å^−^^3^
174 parameters	Δρ_min_ = −0.18 e Å^−^^3^
Primary atom site location: structure-invariant direct methods	Extinction correction: none

### Special details

**Table:**

Geometry. All e.s.d.'s (except the e.s.d. in the dihedral angle between two l.s. planes) are estimated using the full covariance matrix. The cell e.s.d.'s are taken into account individually in the estimation of e.s.d.'s in distances, angles and torsion angles; correlations between e.s.d.'s in cell parameters are only used when they are defined by crystal symmetry. An approximate (isotropic) treatment of cell e.s.d.'s is used for estimating e.s.d.'s involving l.s. planes.
Refinement. Refinement of *F*^2^ against ALL reflections. The weighted *R*-factor *wR* and goodness of fit *S* are based on *F*^2^, conventional *R*-factors *R* are based on *F*, with *F* set to zero for negative *F*^2^. The threshold expression of *F*^2^ > σ(*F*^2^) is used only for calculating *R*-factors(gt) *etc*. and is not relevant to the choice of reflections for refinement. *R*-factors based on *F*^2^ are statistically about twice as large as those based on *F*, and *R*- factors based on ALL data will be even larger.

### Fractional atomic coordinates and isotropic or equivalent isotropic displacement parameters (Å^2^)

**Table:**

	*x*	*y*	*z*	*U*_iso_*/*U*_eq_	
C1	0.5949 (3)	0.12139 (11)	0.32324 (19)	0.0767 (6)	
H1	0.5483	0.1411	0.2427	0.092*	
C2	0.6689 (3)	0.06296 (12)	0.3206 (2)	0.0898 (7)	
H2	0.6696	0.0428	0.2382	0.108*	
C3	0.7414 (4)	0.03451 (11)	0.4398 (3)	0.0976 (7)	
H3	0.7902	−0.0050	0.4379	0.117*	
C4	0.7420 (4)	0.06412 (12)	0.5614 (2)	0.0936 (7)	
H4	0.7959	0.0450	0.6412	0.112*	
C5	0.6643 (3)	0.12151 (11)	0.56657 (19)	0.0777 (6)	
H5	0.6608	0.1410	0.6497	0.093*	
C6	0.5907 (3)	0.15033 (9)	0.44679 (17)	0.0638 (5)	
C7	0.6507 (3)	0.25841 (8)	0.49729 (18)	0.0680 (5)	
C8	0.5528 (3)	0.31031 (9)	0.42952 (16)	0.0657 (5)	
C9	0.3701 (3)	0.29191 (11)	0.34882 (16)	0.0677 (5)	
C10	0.2076 (3)	0.32926 (10)	0.26424 (18)	0.0837 (6)	
H10A	0.1999	0.3158	0.1715	0.126*	
H10B	0.2536	0.3705	0.2689	0.126*	
H10C	0.0646	0.3256	0.2978	0.126*	
C11	0.1418 (3)	0.19847 (10)	0.3420 (2)	0.0868 (6)	
H11A	0.0685	0.2043	0.4232	0.130*	
H11B	0.1695	0.1565	0.3300	0.130*	
H11C	0.0497	0.2133	0.2650	0.130*	
C12	0.8174 (3)	0.38194 (9)	0.49480 (17)	0.0709 (5)	
H12	0.8987	0.3517	0.5407	0.085*	
C13	0.9078 (3)	0.44231 (9)	0.49655 (17)	0.0647 (5)	
C14	0.7891 (3)	0.49092 (10)	0.44065 (18)	0.0758 (6)	
H14	0.6464	0.4852	0.4000	0.091*	
C15	0.8790 (3)	0.54686 (9)	0.44463 (19)	0.0782 (6)	
H15	0.7957	0.5787	0.4070	0.094*	
N1	0.5147 (2)	0.21053 (8)	0.45312 (14)	0.0699 (5)	
N2	0.3520 (2)	0.23112 (8)	0.35370 (14)	0.0709 (5)	
N3	0.6298 (2)	0.36905 (8)	0.43221 (14)	0.0692 (4)	
O1	0.8196 (2)	0.25210 (6)	0.57570 (14)	0.0871 (5)	

### Atomic displacement parameters (Å^2^)

**Table:**

	*U*^11^	*U*^22^	*U*^33^	*U*^12^	*U*^13^	*U*^23^
C1	0.0660 (11)	0.1017 (18)	0.0610 (13)	−0.0126 (11)	−0.0015 (9)	−0.0058 (11)
C2	0.0811 (14)	0.1034 (19)	0.0843 (16)	−0.0086 (13)	0.0044 (12)	−0.0195 (14)
C3	0.0867 (15)	0.0909 (17)	0.113 (2)	−0.0014 (12)	−0.0011 (14)	−0.0023 (16)
C4	0.0888 (15)	0.107 (2)	0.0824 (17)	0.0020 (13)	−0.0072 (12)	0.0115 (14)
C5	0.0653 (11)	0.1053 (18)	0.0605 (13)	−0.0029 (11)	−0.0064 (9)	−0.0020 (11)
C6	0.0466 (9)	0.0866 (15)	0.0563 (11)	−0.0086 (9)	−0.0051 (8)	−0.0048 (10)
C7	0.0526 (9)	0.0930 (15)	0.0554 (11)	−0.0021 (9)	−0.0110 (8)	−0.0130 (10)
C8	0.0527 (9)	0.0910 (15)	0.0509 (10)	0.0064 (10)	−0.0094 (8)	−0.0077 (9)
C9	0.0487 (9)	0.1015 (17)	0.0505 (10)	0.0041 (10)	−0.0086 (8)	−0.0064 (10)
C10	0.0628 (11)	0.1145 (17)	0.0696 (13)	0.0125 (10)	−0.0170 (9)	−0.0040 (11)
C11	0.0511 (10)	0.1288 (18)	0.0764 (13)	−0.0119 (10)	−0.0170 (9)	−0.0014 (12)
C12	0.0620 (11)	0.0919 (15)	0.0562 (11)	0.0073 (10)	−0.0099 (9)	−0.0055 (10)
C13	0.0590 (10)	0.0832 (14)	0.0497 (10)	0.0020 (9)	−0.0070 (8)	−0.0066 (9)
C14	0.0555 (10)	0.0959 (17)	0.0716 (12)	0.0021 (10)	−0.0187 (9)	−0.0014 (11)
C15	0.0643 (11)	0.0872 (16)	0.0781 (13)	0.0062 (11)	−0.0210 (10)	0.0021 (11)
N1	0.0542 (8)	0.0945 (13)	0.0571 (9)	0.0002 (8)	−0.0174 (7)	−0.0081 (8)
N2	0.0477 (8)	0.1039 (14)	0.0570 (9)	0.0000 (8)	−0.0175 (7)	−0.0055 (8)
N3	0.0579 (9)	0.0914 (13)	0.0557 (9)	0.0025 (8)	−0.0085 (7)	−0.0079 (8)
O1	0.0706 (8)	0.0991 (11)	0.0832 (9)	0.0021 (7)	−0.0401 (7)	−0.0085 (7)

### Geometric parameters (Å, °)

**Table:**

C1—C2	1.379 (3)	C9—C10	1.488 (2)
C1—C6	1.382 (2)	C10—H10A	0.9600
C1—H1	0.9300	C10—H10B	0.9600
C2—C3	1.373 (3)	C10—H10C	0.9600
C2—H2	0.9300	C11—N2	1.464 (2)
C3—C4	1.370 (3)	C11—H11A	0.9600
C3—H3	0.9300	C11—H11B	0.9600
C4—C5	1.366 (3)	C11—H11C	0.9600
C4—H4	0.9300	C12—N3	1.279 (2)
C5—C6	1.384 (2)	C12—C13	1.453 (3)
C5—H5	0.9300	C12—H12	0.9300
C6—N1	1.422 (2)	C13—C14	1.388 (2)
C7—O1	1.2360 (19)	C13—C15^i^	1.391 (2)
C7—N1	1.395 (2)	C14—C15	1.361 (2)
C7—C8	1.438 (2)	C14—H14	0.9300
C8—C9	1.369 (2)	C15—C13^i^	1.391 (2)
C8—N3	1.390 (2)	C15—H15	0.9300
C9—N2	1.361 (2)	N1—N2	1.4056 (17)
			
C2—C1—C6	119.3 (2)	H10A—C10—H10B	109.5
C2—C1—H1	120.4	C9—C10—H10C	109.5
C6—C1—H1	120.4	H10A—C10—H10C	109.5
C3—C2—C1	120.0 (2)	H10B—C10—H10C	109.5
C3—C2—H2	120.0	N2—C11—H11A	109.5
C1—C2—H2	120.0	N2—C11—H11B	109.5
C4—C3—C2	120.2 (2)	H11A—C11—H11B	109.5
C4—C3—H3	119.9	N2—C11—H11C	109.5
C2—C3—H3	119.9	H11A—C11—H11C	109.5
C5—C4—C3	120.7 (2)	H11B—C11—H11C	109.5
C5—C4—H4	119.7	N3—C12—C13	122.21 (17)
C3—C4—H4	119.7	N3—C12—H12	118.9
C4—C5—C6	119.3 (2)	C13—C12—H12	118.9
C4—C5—H5	120.4	C14—C13—C15^i^	117.43 (17)
C6—C5—H5	120.4	C14—C13—C12	122.44 (16)
C1—C6—C5	120.5 (2)	C15^i^—C13—C12	120.13 (17)
C1—C6—N1	120.73 (17)	C15—C14—C13	120.76 (16)
C5—C6—N1	118.80 (17)	C15—C14—H14	119.6
O1—C7—N1	122.90 (17)	C13—C14—H14	119.6
O1—C7—C8	131.90 (17)	C14—C15—C13^i^	121.81 (17)
N1—C7—C8	105.18 (15)	C14—C15—H15	119.1
C9—C8—N3	123.15 (18)	C13^i^—C15—H15	119.1
C9—C8—C7	108.00 (18)	C7—N1—N2	109.12 (15)
N3—C8—C7	128.68 (15)	C7—N1—C6	123.38 (14)
N2—C9—C8	109.98 (16)	N2—N1—C6	119.21 (14)
N2—C9—C10	121.68 (16)	C9—N2—N1	107.21 (13)
C8—C9—C10	128.3 (2)	C9—N2—C11	124.40 (15)
C9—C10—H10A	109.5	N1—N2—C11	116.47 (16)
C9—C10—H10B	109.5	C12—N3—C8	120.28 (16)
			
C6—C1—C2—C3	−1.3 (3)	C13—C14—C15—C13^i^	0.4 (3)
C1—C2—C3—C4	−0.5 (3)	O1—C7—N1—N2	−173.36 (16)
C2—C3—C4—C5	2.1 (3)	C8—C7—N1—N2	4.94 (18)
C3—C4—C5—C6	−2.0 (3)	O1—C7—N1—C6	−25.5 (3)
C2—C1—C6—C5	1.4 (3)	C8—C7—N1—C6	152.81 (15)
C2—C1—C6—N1	−179.78 (16)	C1—C6—N1—C7	−114.23 (18)
C4—C5—C6—C1	0.3 (3)	C5—C6—N1—C7	64.6 (2)
C4—C5—C6—N1	−178.63 (17)	C1—C6—N1—N2	30.6 (2)
O1—C7—C8—C9	177.28 (19)	C5—C6—N1—N2	−150.49 (15)
N1—C7—C8—C9	−0.80 (19)	C8—C9—N2—N1	6.81 (18)
O1—C7—C8—N3	2.0 (3)	C10—C9—N2—N1	−173.58 (15)
N1—C7—C8—N3	−176.11 (16)	C8—C9—N2—C11	147.84 (16)
N3—C8—C9—N2	171.85 (15)	C10—C9—N2—C11	−32.6 (2)
C7—C8—C9—N2	−3.78 (19)	C7—N1—N2—C9	−7.32 (18)
N3—C8—C9—C10	−7.7 (3)	C6—N1—N2—C9	−156.74 (15)
C7—C8—C9—C10	176.65 (17)	C7—N1—N2—C11	−151.89 (16)
N3—C12—C13—C14	5.7 (3)	C6—N1—N2—C11	58.7 (2)
N3—C12—C13—C15^i^	−174.14 (16)	C13—C12—N3—C8	177.92 (15)
C15^i^—C13—C14—C15	−0.4 (3)	C9—C8—N3—C12	−170.27 (17)
C12—C13—C14—C15	179.78 (18)	C7—C8—N3—C12	4.4 (3)

Symmetry codes: (i) −*x*+2, −*y*+1, −*z*+1.

### Hydrogen-bond geometry (Å, °)

**Table:**

*D*—H···*A*	*D*—H	H···*A*	*D*···*A*	*D*—H···*A*
C11—H11C···O1^ii^	0.96	2.36	3.321 (2)	179
C11—H11A···O1^iii^	0.96	2.47	3.375 (3)	157
C12—H12···O1	0.93	2.30	3.002 (2)	132

Symmetry codes: (ii) *x*−1, −*y*+1/2, *z*−1/2; (iii) *x*−1, *y*, *z*.
